# Association of *IL-6* G-174C (rs1800795) variant with the susceptibility to hepatocellular carcinoma in patients with chronic hepatitis

**DOI:** 10.1186/s43046-024-00238-y

**Published:** 2024-10-21

**Authors:** Eman H. Abuelnadar, Lamiaa M. Ramadan, Hanaa Elsayed Shahin, Saleha Y. M. Alakilli, Eman Wahsh, Nanis S. El-Beltagy, Eman T. Salem, Abdelrahman S. Hatata, Afaf M. El-Said, Maha Alhelf

**Affiliations:** 1 Department of Laboratories, Mansoura University, Children Hospital, Mansoura, Egypt; 2 Department of Biochemistry, Biotechnology Research Institute, National Research Centre, Cairo, Egypt; 3 Nursing Department, College of Applied Medical Sciences, Jouf University, ElQurayyat, Saudi Arabia; 4 Department of Maternity and Newborn Health Nursing, Health Nursing, Menoufia University, Menoufia, Egypt; 5 Department of Biological Sciences, Faculty of Science, King Abdulaziz University, Jeddah, Saudi Arabia; 6 Department of Pharmacology & Toxicology, Faculty of Pharmacy, Sinai University, Arish Branch, Al-Arish, North Sinai 45511 Egypt; 7 Mansoura University Children’s Hospital, Mansoura, Egypt; 8 Department of Basic Sciences, Faculty of Physical Therapy, Horus University-Egypt, New Damietta, 34518 Egypt; 9 Mansoura Manchester Program, Faculty of Medicine, Mansoura University, Mansoura, Egypt; 10 Genetic Unit, Faculty of Medicine, Mansoura University, Mansoura, Egypt; 11 Biotechnology School, Nile University, Giza, Egypt; 12 Medical Biochemistry and Molecular Biology Department, Faculty of Medicine, Cairo University, Cairo, Egypt

**Keywords:** HCC, Viral hepatitis, IL-6, Polymorphism

## Abstract

**Aim:**

An ineffective immune response resulting from dysregulation of cytokine production might encourage viral persistence and cause chronic viral hepatitis to worsen. This study examined the relationship between alterations in interleukin-6 (IL-6) levels and the *IL-6* − 174 G > C (rs1800795) polymorphism, as well as how this polymorphism affects the development and progression of chronic hepatitis brought on by hepatitis B (HBV) and hepatitis C (HCV) into hepatocellular carcinoma (HCC).

**Patients and methods:**

Whole blood samples from 126 Egyptian patients with HCC (111 with HCV and 15 with HBV), as well as 126 age- and sex-matched healthy individuals, were used to extract DNA. Using PCR-based allele-specific amplification (ASA), the existence of the *IL-6* G-174C polymorphism was investigated. Additionally, each participant's serum IL-6 levels were determined using an enzyme-linked immunosorbent assay (ELISA).

**Results:**

The primary observations revealed that HCC patients had greater serum levels of IL-6 compared to the control groups (*p* < 0.001). Patients with the variant (CG and GG) genotype in the HCC group were found to have more disease severity indicated by higher levels of alpha-fetoprotein (AFP) and a higher ascites grade, as well as increased inflammatory activity as defined by higher levels of IL-6 and C-reactive protein (CRP) (*p* < 0.001 for both) in comparison to patients with the wild-type (CC) genotype (*p* < 0.001 and *p* = 0.002, respectively).

**Conclusion:**

The rs1800795 SNP in the *IL-6* gene was associated with increased inflammatory activity and high levels of IL-6, indicating that this SNP may play a role in the development of HCC in Egyptian patients with chronic viral hepatitis.

**Supplementary Information:**

The online version contains supplementary material available at 10.1186/s43046-024-00238-y.

## Introduction

Liver cancer is the sixth most common disease globally, accounting for over 900,000 new cases annually (about 4.7% of all cases). Despite widely recognized risk factors, 8.3% of cancer-related deaths worldwide are attributed to it, making it the third most common cause of cancer-related mortality worldwide [1, 2].

In primary liver cancer, hepatocellular carcinoma (HCC) makes up over 80% of cases. In Egypt, HCV and HBV patients are more likely to have HCC, which is the fourth most prevalent malignancy [3, 4]. Three to four times as many males as females are found to have HCC. Lifestyle choices such as smoking and alcohol usage that raise the risk of HCC could be the reason for this gender disparity. Nonetheless, as men are more likely than women to develop HCC, X-linked chromosome genetic factors and sex hormones may be important in the pathogenesis of the disease [5].

The immune system typically fails to eradicate HCV and HBV infections in their mature phases, which results in lifelong host-virus interaction and chronic liver inflammation [6]. Interleukins and other cytokines produced by macrophages and lymphocytes control the antiviral response. Immune cells such as B cells, T cells, macrophages, and fibroblasts all produce IL-6, a pro-inflammatory cytokine with pleiotropic effects on hematopoiesis, inflammation, and immunological response. The primary role of IL-6 is to modulate the hepatic response to systemic inflammation and infections [7]. There is mounting evidence that the development of cancer is aided by persistent inflammation. High levels of IL-6 have been linked to a number of clinical illnesses, including neoplastic and inflammatory diseases, and liver diseases, including chronic viral hepatitis [8], alcoholic liver disease [9], and liver cirrhosis [10].

The balance between pro- and anti-inflammatory cytokines may be influenced by genetic factors, which may have an impact on viral infection severity, clinical prognosis, and an individual’s susceptibility to HCC. This is the subject of growing research. The ratio of pro-to-anti-inflammatory cytokines may change how a disease progresses and how beneficial antiviral treatment is [11].

HCC progression on top of chronic hepatitis has been linked to single nucleotide polymorphisms (SNPs) in a number of interleukin genes [12]. Polymorphisms in the *IL-6* gene appear to be involved in the pathogenesis of some immune-mediated diseases. These SNPs cause individuals with chronic HCV and HBV hepatitis, as well as alcoholics, to respond differently to HCC treatment and have enhanced liver cell proliferation [11, 13, 14].

The *IL-6* G-174C (rs1800795) SNP has been linked in several studies to the transcription rate and, consequently, to the regulation of plasma IL-6 levels [15]. This SNP has two phenotypes: the low-producer phenotype, which included the − 174 CC genotype, and the high-producer phenotype, which included the − 174 GG and − 174 GC genotypes and was marked by increased plasma IL-6 levels [13, 15]. In our study, we investigated the impact of the *IL-6* G-174C SNP on the circulatory level of IL-6 and its correlation with HCC risk among Egyptian patients with chronic HCV or HBV infections.

## Patients and methods

### Patients

After the approval (PT-2024–001) from the Horus University Ethics Committee, this case–control study was carried out on 126 patients with primary HCC diagnosed at the Oncology Centre Mansoura University, Mansoura, Egypt, along with126 healthy volunteers who were matched for age and sex as a control group. For the diagnosis of HCC, pathological and histological screenings were combined with medical imaging, such as CT or MRI. Patients with HCC who tested positive for HBV or HCV were included, while individuals with HIV infection, autoimmune illnesses, renal failure, or any other type of cancer were not eligible. As per the International Ascites Club [16], there are three categories for ascites grades: mild (I), moderate (II), and massive (III). The study was conducted from March 2023 to January 2024. Written informed consent was completed by every participant in this research.

### Methods

Five milliliters of blood were collected from each participant and each sample was divided into two aliquots: two milliliters were put into sterile tubes containing EDTA for the hematological and molecular analyses, while the remaining blood was allowed to clot, centrifuged for ten minutes at 4000 rounds per minute, and the serum was then separated and kept at − 20 °C for additional biochemical analyses.

#### Genotyping of IL-6 G-174C (rs1800795)

Following the manufacturer's instructions, whole blood samples from each participant were used to extract genomic DNA using a commercial extraction kit (QIAamp ® DNA Extraction Kit, Cat. No. #51,106, QIAGEN, Hilden, Germany). Following extraction, the NanoDropTM 1000 Spectrophotometer (NanoDrop Tech., Inc. Wilmington, USA) was used to examine the quality of the extracted DNA.

The analysis of *IL-6* G-174C (rs1800795) genotyping and amplification was conducted by the use of allele-specific amplification-PCR (ASA), using the following primers: F1-wild-type (5′-CCTATTGTGTCTTGCC-3′), F2-variant (5′-CCCTAGTTGTGTCTTGCG-3′), and R-common (5′-GAGCTTCTCTTTCGTTCC-3′), as previously reported by Elsaid et al. [12]. Thermo Fisher Scientific’s Thermal Cycler (Applied Biosystem, Waltham, USA) was used to run the PCR. The thermal cycler was used according to the cycling program, as shown in Table S1. Lastly, the 2.5% Agarose gel and 1X Tris–borate-EDTA (TBE) buffer electrophoresis at 200 V for 30 min was used to directly analyze the PCR products of the *IL-6* G-174C amplification (234 bp). The samples were then stained with ethidium bromide (500 mg/L) and observed under an ultraviolet trans-illuminator.

#### Biochemical and hematological measurements

Biochemical measurements, including serum ALT (Biomed Diagnostics, Germany), AST (Biomed Diagnostics, Germany), total bilirubin (BioVision, Inc., USA), albumin (Biomed Diagnostics, Germany), creatinine (Abcam, USA), and CRP (Linear Chemicals, Spain), were assessed using colorimetric assay kits according to the constructor’s guidelines. Utilizing an Automated Hematology Analyzer Abbott Cell-Dyn Ruby, USA, a complete blood count (CBC) was performed. Additionally, the ELISA kit (Cat. No. ab193765, Abcam, USA) was used to measure the serum level of alpha-fetoprotein (AFP) and another ELISA kit (Cat. No. E0090Hu, Bioassay Technology Laboratory, China) was used to measure serum IL-6 level.

#### Statistical analysis

The study published by Elsaid et al. [12] and G*Power (Version 3.1.9.2) [17] were used to compute the sample size. To verify normality, the Kolmogorov–Smirnov test was employed. Normal distributed data was described by the mean ± standard deviation (SD), non-normally distributed data by the median (minimum and maximum), and qualitative data by percentages and numerical values. The Student *t*-test and the one-way ANOVA test were used to handle the parametric data, while the Mann–Whitney and Kruskal–Wallis procedures were used to analyze the non-parametric data. To investigate the association between the qualitative variables, the following tests were used: Fisher exact, Monte Carlo, and chi-square (*χ*2). To determine how strongly two quantitative variables were associated, correlation analysis (*r*) was employed. A helpful tool for assessing the sensitivity and specificity of quantitative diagnostic measures is the ROC curve. AUC of 0.9 shows high accuracy; 0.7–0.9 denotes moderate accuracy; 0.5–0.7 denotes low accuracy; and 0.5 is a result of chance.

The cutoff point for significance was set at *p* < 0.05). Binary logistic regression was used to determine the odds ratio (OR) and confidence intervals (CI) of 95%.

## Results

Table 1 reads the primary clinical, laboratory, and demographic data for the groups under investigation. In the current study, 126 Egyptian patients with HCC—80.2% men and 19.8% women—with a mean age of 57.74 ± 8.47 years and a range of 40 to 71 years were paired with 126 healthy persons based on age and sex. We could not identify any appreciable differences between the two groups in terms of age or gender.

**Table 1:** The main characteristics and lab measurements of the studied groups

Parameters	HCC (*n* = 126)	Control(*n* = 126)	*p* value
Age (years),	57.74 ± 8.47	56.87 ± 8.85	= 0.42
Gender (M/F), *n* (%)	101 (80.2%)	91 (72.2%)	= 0.14
	25(19.8%)	35 (27.8%)	
Positive HCV, *n* (%)	111 (88%)	0 (0%)	< 0.001^*^
Positive HBV, *n* (%)	15 (12%)	0 (0%)	< 0.001^*^
Positive smoking, *n* (%)	36 (28.6%)	15 (12%)	< 0.001^*^
Positive ascites,* n (%)*	85 (67.5%)	–	–
Ascites grades			
I, *n* (%)	35 (41.2%)		
II, *n* (%)	29 (34.1%)		
III, *n* (%)	21 (24.7%)		
Biochemical measurements			
ALT (U/L)	41 (23–262)	28 (20–40)	< 0.001^*^
AST (U/L)	63.5 (24–297)	30 (15–38)	< 0.001^*^
Total bilirubin (mg/dl)	3.5 (0.9–15.8)	0.78 (0.45–1.07)	< 0.001^*^
Albumin (g/dl)	2.64 ± 0.72	4.29 ± 0.31	< 0.001^*^
Creatinine (mg/dl)	1.1 (0.7–3.8)	0.91 (0.7–1.22)	< 0.001^*^
AFP (ng/ml)	229 (7–1490)	3 (0.09–6.1)	< 0.001^*^
CRP (mg/dl)	23.5 (0.8–95)	2 (0.16–5.95)	< 0.001^*^
Hematological measurements			
RBCs (× 10^12^/L)	3.54 ± 0.75	4.52 ± 0.59	< 0.001^*^
WBCs (× 10^9^/L)	10.26 ± 5.69	7.85 ± 1.23	< 0.001^*^
Hemoglobin (g/dl)	10.51 ± 2.04	13.09 ± 0.88	< 0.001^*^
Platelets (× 10^9^/L)	141 (77–534)	274 (221–322)	< 0.001^*^

Student’s *t*-test, Mann–Whitney *U* test, and chi-square test were applied

^*^Significant at *p < *0.05

In HCC group, 111 patients (88%) were positive for HCV, while 15 (12%) were positive for HBV. It was noticed that 85 cases (67.5%) had ascites, with 35 (41.2%) having ascites grade I, 29 (34.1%) having grade II, and 21 (24.7%) having grade III. HCC patients had significantly elevated levels of ALT, AST, total bilirubin, creatinine, and CRP levels (*p* < 0.001 for all), as well as significantly decreased RBCs, platelets count, hemoglobin, and albumin levels compared to the control group (*p* < 0.001 for all).

**Fig. 1:**
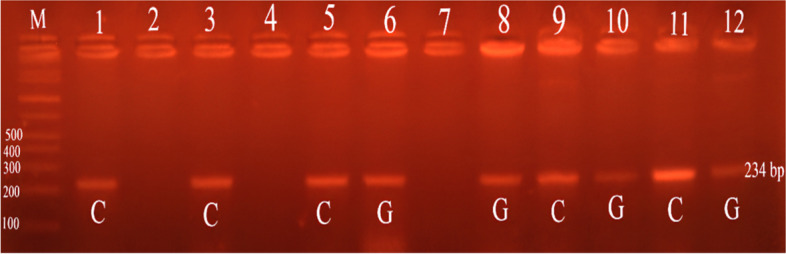
Agarose gel electrophoresis for *IL6* G-174C polymorphism in the studied groups. Lane (M) represents DNA marker (100 bp thermo scientific); lanes 1, 2, 3, and 4 displayed (CC genotype, C allele at 234 bp); lanes 5, 6, 9, 10, 11, and 12 showed (CG genotype at 234 bp); lanes 7 and 8 showed (GG genotype, G allele at 234 bp)

Three genotypes were identified based on the ASA-PCR investigation of the *IL-6* G-174C polymorphism: wild-type (CC), heterozygous carrier (CG), and homozygous variant (GG). Upon separation on an agarose gel, the PCR outcome was displayed in Fig. 1.

The first PCR reaction showed the CC genotype as one band at 234 bp, the second PCR reaction showed the GG genotype as one band at 234 bp, and the third PCR reaction showed the CG genotype as two bands at 234 bp in both reactions.

**Fig. 2:**
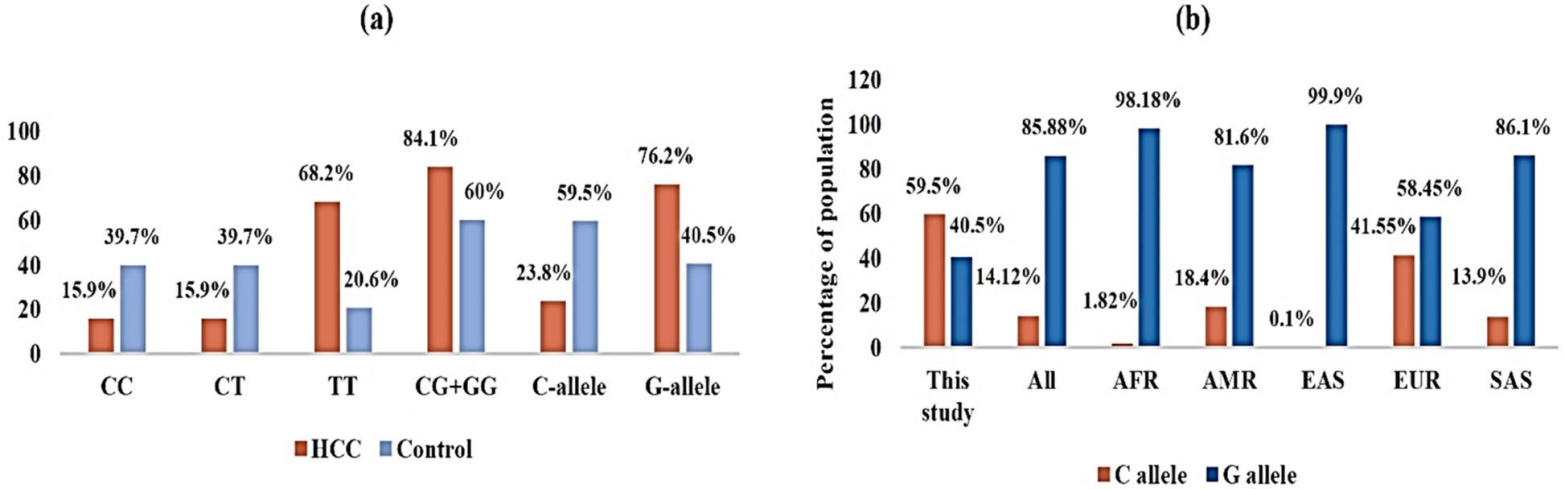
**a** Genotypic and allelic frequencies of the *IL-6* G-174C variant among studied groups. **b** Allelic frequencies of the *IL-6* G-174C variant in the current work in comparison to different populations based on the 1000 Genome project phase 3. (https://www.internationalgenome.org/). AFR: Africa, AMR: America, EAS: East Asia, EUR: Europe, SAS: South Asia

The frequency of the G-allele was found to be 76.2% in patients and 40.5% in healthy controls, while the C-allele is shared by 23.8% of patients and 59.5% of healthy people. The frequency of the wild-type genotype (CC) was 15.9% in patients and 39.7% in healthy controls (Fig. 2). Upon combining different genetic association models, HCC patients showed a strong link with the *IL-6* G-174C polymorphism. The co-dominant model (GG vs. CC, OR = 8.27, *p* < 0.001), the dominant model (CG + GG vs. CC, OR = 3.48, *p* < 0.001), the recessive model (GG vs. CC + CG, OR = 8.27, *p* < 0.001), and the allelic model (G-allele vs. C-allele, OR = 4.7, *p* < 0.001), as shown in Table 2. The bioinformatics outlines of the *IL-6* gene are presented in Figure S1.

**Table 2:** Comparison of *IL-6*-G174C polymorphism among HCC patients and control groups

*IL-6* G-174C		HCC *n* (%)		Control *n* (%)		*p* value	OR (95% CI)	
		*N*	%	*n*	%			
Codominant	CC	20.0	15.9%	50.0	39.7%		Reference	
	CG	20.0	15.9%	50.0	39.7%	< 0.001^*^	1.0	0.48–2.08
	GG	86.0	68.2%	26.0	20.6%	< 0.001^*^	8.27	4.19–16.31
Dominant model	CC	20.0	15.9%	50.0	39.7%		Reference	
	CG + GG	106.0	84.1%	76.0	60.3%	< 0.001^*^	3.48	1.93–6.28
Recessive model	CC + CG	40.0	31.7%	100.0	79.4%		Reference	
	GG	86.0	68.3%	26.0	20.6%	< 0.001^*^	8.27	4.67–14.65
Allelic model	C	60.0	23.8%	150.0	59.5%		Reference	
	G	192.0	76.2%	102.0	40.5%	< 0.001^*^	4.7	3.2–6.9

Chi-square test was applied. *OR* odds ratio, *CI* confidence interval

^*^Statistically significant (if *p* < 0.05)

The CC, CG, and GG genotypes for the *IL-6* G-174C variant were compared regarding the demographic, clinical characteristics, and lab measurements of HCC patients, as shown in Table 3. The HCC patients in the (CG) and (GG) groups had considerably higher AFP and CRP levels than the patients in the (CC) group (*p* < 0.001 for both). Additionally, the percentages of patients with higher ascites grades were greater in the (CG) and (GG) groups compared to those in the (CC) group (*p* < 0.001).

**Table 3:** Association of *IL-6* G-174C variant with the main characteristics and lab measurements of the HCC patients

Parameters	*CC* *n* = *20*	*CG n* = *20*	*GG n* = *86*	*p* value	*p* value within groups
Age (years)	61.15 ± 7.93	57.75 ± 7.37	59.95 ± 8.7	= 0.14	–
Gender (M/F)	16/4	17/3	68/18	= 0.83	–
Smoking status (yes/no)	4/16	6/14	26/60	= 0.65	-
Ascites; *n* (%)					
Absent	17(85%)	9 (45%)	15 (17.4%)	< 0.001^*^	*p*_1 _= 0.05 *p*_2 _< 0.001^*^ *p*_3 _= 0.06
I	1 (5%)	5 (25%)	29 (33.7%)		
II	2(10%)	4 (20%)	23 (26.7%)		
III	0 (0%)	2 (10%)	19 (22.2%)		
Biochemical measurements					
ALT (U/L)	45.5 (23–262)	40 (28–102)	41 (23–214)	= 0.83	–
AST (U/L)	53.5 (28–294)	89 (28–293)	63.5 (23–297)	= 0.88	–
Total bilirubin (mg/dl)	3.5 (1.2–15)	3.5 (1.1–15.8)	3.2 (0.9–14)	= 0.37	–
Albumin (g/dl)	2.64 ± 0.73	2.6 ± 0.78	2.7 ± 0.72	= 0.97	–
Creatinine (mg/dl)	0.8 (0.71–3.8)	1.05 (0.73–3.6)	1.25 (0.7–3.72)	= 0.27	–
AFP (ng/ml)	96.5 (7–258)	250 (8.2–729)	400 (7–1490)	< 0.001^*^	*p*_1 _= 0.013^*^ *p*_2 _= 0.011^*^ *p*_3 _= 0.9
CRP (mg/dl)	8.7 (7–258)	16.9 (13.3–20.8)	32 (1–95)	< 0.001^*^	*p*_1 _= 0.001^*^ *p*_2 _< 0.001^*^ *p*_3 _< 0.001^*^
Hematological measurements					
RBCs (× 10^12^/L)	3.69 ± 0.66	3.55 ± 0.62	3.51 ± 0.8	= 0.63	–
WBCs (× 10^9^/L)	11.85 ± 5.92	11.49 ± 5.5	9.6 ± 5.63	= 0.63	–
Hemoglobin (g/dl)	10.72 ± 2.22	10.48 ± 1.92	10.46 ± 2.04	= 0.88	–
Platelets (× 10^9^/L)	177 (79–450)	177 (89–451)	121.5 (77–534)	= 0.18	–

Student’s *t* test, Man-Whitney *U* test, one-way ANOVA test, Kruskal–Wallis test, chi-square test, Fisher exact test and Monte Carlo test were applied

^*^Significant at *p < *0.05, *p*_*1*_ comparison of CC vs. CG, *p*_*2*_ comparison of CC vs. GG, *p*_*3*_ comparison of CG vs. GG

**Fig. 3:**
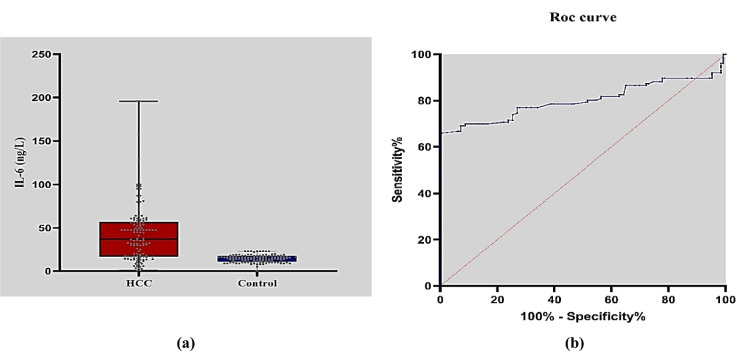
**a** Box and Whisker plot for IL-6 level in the HCC and control groups. **b** ROC curve of IL-6 for the diagnosis of HCC

Serum IL-6 concentration was significantly elevated among HCC patients 37 (1–195.8 ng/L) compared to controls 15 (0.5–23 ng/L) (*p* < 0.001) (Fig. 3a). Figure 3b showed that the best cutoff value of IL-6 protein for HCC diagnosis was ≥ 17.0 ng/L with area under the curve = 0.801, sensitivity = 74.6% and specificity = 73%.

**Fig. 4:**
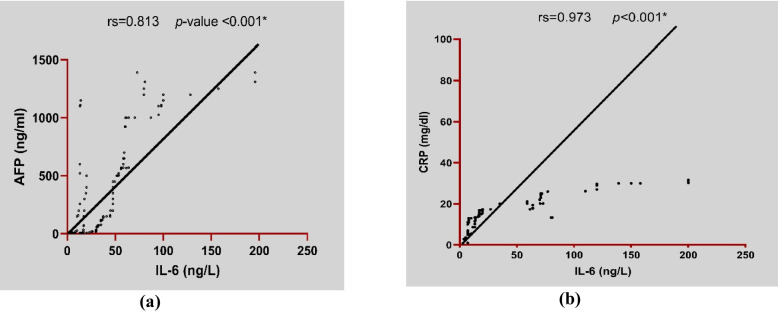
Spearman’s rank correlation coefficient values evaluate the relationships between the IL-6 level and **a** AFP; **b** CRP in the HCC group

Correlation of serum IL-6 with the studied parameters in the HCC group showed that the concentration of IL-6 was significantly higher in patients with (GG) and (CG) genotypes 47.5 (1–195.8 ng/L) and 17.85 (13.3–73 ng/L), respectively than in patients with (CC) genotype 11.75 (2.8–81 ng/L) (*p* < 0.001). IL-6 was also significantly higher in the patients presented with ascites compared to those without ascites (*p* = 0.002), as shown in Table 4. A positive significant correlation was found in the HCC group between IL-6 levels and each of AFP and CRP levels (*p* < 0.001 for both), as shown in Table S2, Fig. 4a, b.

**Table 4:** Association between IL-6 levels and some characteristics of patients with HCC

Parameter		IL-6 (ng/L), Median (range)	*p* value		
Gender	Male	36.5 (2.8–195.8)	*p* = 0.57		
	Female	45 (1–195)			
Ascites	Absent	17.3 (2.8–81)	*p* = 0.002^*^		
	I	47.5 (1–100)			
	II	47.5 (3.6–193)			
	III	56.2 (6–61.5)			
Genotypes	CC	11.75 (2.8–81)	*p* < 0.001^*^		
	CG	17.85 (13.3–73)	*p*_1_	*p*_2_	*p*_3_
	GG	47.5 (1–195.8)	= 0.001^*^	< 0.001^*^	< 0.001^*^

Mann–Whitney *U* test and Kruskal–Wallis test were applied

^*^Significant at *p < *0.05, *p*_*1*_ comparison of CC vs. CG, *p*_*2*_ comparison of CC vs. GG, *p*_*3*_ comparison of CG vs. GG

## Discussion

The occurrence and tumorigenicity of hepatocellular carcinoma (HCC), a frequent malignant tumor, involve multistep and multifactor interactions [18]. Numerous investigations have demonstrated that patients with HCV and HBV infection had higher serum levels of IL-6. Such hepatitis viruses cause inflammation in liver tissue. Many studies have demonstrated the critical role that HCV and HBV infections play in the development of HCC through the IL-6/STAT3 pathway [19–21].

In order for IL-6 to play a part in anti-apoptosis, angiogenesis, proliferation, invasion, metastasis, and drug resistance of cancer cells, it must first bind to the IL-6 receptor (IL-6R), which in turn activates the Janus kinase (JAK) linked to the receptor, stimulating phosphorylation and activating signal transducer and activator of transcription 3 (STAT3) to initiate downstream signals [22].

The IL-6R system is mainly composed of the IL-6 ligand binding chain and signal transduction chain, namely, gp130. Numerous cells, including hepatocytes, monocytes, macrophages, and neutrophils are known to produce IL-6R, which is classified into two categories: soluble IL-6R (sIL-6R) and membrane-bound IL-6R (mIL-6R). mIL-6R is found on the surface of cells, and sIL-6R is created either directly through splicing mRNA during the translation phase or indirectly through protein hydrolysis of mIL-6R on the membrane [23]. IL-6 binds to mIL-6R on the membrane, causing dimerization, which initiates signal transduction in the classical signaling transduction pathway. IL-6 first connects with sIL-6R during signal transduction, and the complex subsequently binds with membrane gp130 [24, 25].

Several investigations have been conducted to examine the connection between different genetic factors and the vulnerability of HCC in hosts infected with HBV and HCV in different populations. The most often documented gene associated with the development of HCC in patients with chronic viral hepatitis is *IL-6*. It has been documented that variations in the *IL-6* gene can affect the course of the histology and the clinical results of individuals suffering from persistent viral hepatitis [26–28]. Thus, we intended to determine the possible association of *IL-6* gene (rs1800795) SNP and HCC development among Egyptian patients with chronic C and B hepatitis.

ASA-PCR was used to detect the gene polymorphism, and the results showed that the variant G-allele was much more common in HCC patients than in controls (*p* < 0.001), with an estimated fivefold increased risk of developing HCC (OR = 4.7). According to our findings, IL-6 levels in HCC patients were significantly higher than in controls.

Regarding the different genotype groups, the CG and GG genotypes were correlated with the inflammatory state of HCC patients. The IL-6 and CRP levels were significantly elevated in patients carrying CG and GG genotypes compared to those carrying CC genotypes (*p* < 0.001). Furthermore, regarding the disease prognosis, higher AFP levels and higher ascites grades were observed in patients with CG and GG genotypes, compared to the CC carriers (*p* < 0.001 and *p* = 0.002, respectively).

The pooled study utilizing continuous data in a meta-analysis of eighteen studies showed higher levels of IL-6 in HCC patients as compared to hepatitis and healthy controls. Compared to healthy controls, IL-6 levels in HCC patients were considerably higher (mean difference = 12.44, 95% CI 9.02–15.85, *p* < 0.001) [29].

Our findings aligned with earlier research on *IL-6* G-174C in individuals with head and neck cancer. In a population-based case–control study of HCC, which involved 230 matched control people and 120 HCC cases, researchers looked at the relationship between *IL-6* gene genetic variants and HCC risk in non-Asian populations. The researchers showed that, out of all the cytokine polymorphisms examined, the GG* IL-6* genotype had the greatest impact on the risk of HCC [30]*.* In a more recent investigation, Falleti et al. examined 219 consecutive patients who had liver transplantation due to liver cirrhosis in order to determine whether *IL-6* polymorphisms could be connected to the development of HCC in these individuals. They discovered a strong correlation between the lack of HCC and the low-producer genotype (− 174 CC) [31].

Another study aimed to examine the blood levels of IL-6 and the frequency of SNPs in the *IL-6* promoter region at position − 174 in a sample of patients with head and neck cancer. They discovered that in HCC patients, IL-6 serum level was greater in the GG genotype than in CC genotypes and that there was a correlation between IL-6 serum levels and G carriers in HCC [32].

Furthermore, according to a meta-analysis by Tian et al., significance was only reached for liver cancer under allelic (OR = 0.74; *P* = 0.001), homozygous genotypic (OR = 0.59; *p* = 0.029), and dominant (OR = 0.67; *P* = 0.004) models, with the – 174C allele conferring a decreased risk. When compared to non-carriers, carriers of the – 174CC genotype (mean difference = – 4.23 pg/ml,* p* < 0.001) and – 174C allele (mean difference = – 3.43 pg/ml, *p* < 0.001) had considerably lower levels of circulating IL-6 [33]. An additional Mendelian randomization analysis revealed a strong correlation between a 12% lower risk of liver cancer and a drop in circulating IL-6 of 1 pg/ml. Liver cancer risk may be correlated with long-term genetically reduced circulating IL-6 levels of cancer [33].

## Conclusion

In conclusion, in individuals with chronic viral hepatitis, our study found a strong correlation between the G-174C (rs1800795) SNP within the *IL-6* gene and HCC susceptibility. In order to use these SNPs as biomarkers in the future for risk stratification of HCC onset, prognosis prediction, and clinical disease progression evaluation, more thorough research will be needed to examine the potential contribution of *IL-6* polymorphisms to HCC progression in affected individuals. Blocking the IL-6/STAT3 signaling pathway can be used to treat HCC because the IL-6/STAT3 signal axis induces an immunosuppressive effect in the tumor microenvironment.

## Supplementary Information

Supplementary Material 1: Figure S1. (a) The IL-6 gene [ENSG00000136244] has some synonyms, including CDF, HGF, HSF, BSF2, BSF-2, IFNB2, and IFN-beta-2. The IL-6 gene is located on the short arm of chromosome number 7 (Ch7p15.3). IL-6 gene contains six exons and five introns. The (rs1800795) is an intronic variant guanine (C) to cytosine (G) replacement at −174 position (−174G/C) with the highest population MAF equals to 0.48 [Data source: Ensembl.org; Human Genome Assembly GRCh38.p13]. (b, c) The IL-6 gene encodes a signaling protein named interleukin-6 (IL-6) that comprises 212 amino acids with a molecular mass of 23,718 Daltons [Data source: Uniprot database (P05231)]. (d) Protein interaction networks recommend that IL-6 is a potent inducer of the acute phase response and has a major function in immune system regulation [Data source: STRING]. (e) The main cellular IL-6 is located within the extracellular space and endoplasmic reticulum [Data source: Cellular compart¬ment database]. Table S1. Thermal cycler program for 894 G>T variant. Table S2. Correlations of IL-6 level with other parameters among HCC group.

## Data Availability

The dataset utilized in the preparation of this study will be available from the corresponding author upon reasonable request.

## Abbreviations

HCC: Hepatocellular carcinoma
AFP: Alpha-fetoprotein
ALT: Alanine transaminase
ASA: Allele-specific amplification
AST: Aspartate transaminase
AUC: Area under the curve
CBC: Complete blood count
CRP: C-reactive protein
EDTA: Ethylene diamine tetra acetic acid
ELISA: Enzyme-linked immunosorbent assay
HBV: Hepatitis B virus
HCV: Hepatitis C virus
IL-6: Interleukin-6
JAKs: Janus kinase
MAF: Minor allele frequency
mIL-6 R: Membrane interleukin-6 receptor
OCMU: Oncology Center Mansoura University
PCR: Polymerase chain reaction
RBCs: Red blood cells
ROC: Receiver operating characteristic
sIL-6R: Soluble interleukin-6 receptor
SNP: Single nucleotide polymorphism
TBE: Tris-borate-EDTA
WBCs: White blood cells
