# Supplementary material for: Association of IL-6 G-174C (rs1800795) variant with the susceptibility to hepatocellular carcinoma in patients with chronic hepatitis
Source: J Egypt Natl Canc Inst. 2024 Oct 21;36:32. doi: 10.1186/s43046-024-00238-y (PMC13313833; doi:10.1186/s43046-024-00238-y)
Supplement: Supplementary file 1 — Supplementary Material 1: Figure S1. (a) The IL-6 gene [ENSG00000136244] has some synonyms, including CDF, HGF, HSF, BSF2, BSF-2, IFNB2, and IFN-beta-2. The IL-6 gene is located on the short arm of chromosome number 7 (Ch7p15.3). IL-6 gene contains six exons and five introns. The (rs1800795) is an intronic variant guanine (C) to cytosine (G) replacement at −174 position (−174G/C) with the highest population MAF equals to 0.48 [Data source: Ensembl.org; Human Genome Assembly GRCh38.p13]. (b, c) The IL-6 gene encodes a signaling protein named interleukin-6 (IL-6) that comprises 212 amino acids with a molecular mass of 23,718 Daltons [Data source: Uniprot database (P05231)]. (d) Protein interaction networks recommend that IL-6 is a potent inducer of the acute phase response and has a major function in immune system regulation [Data source: STRING]. (e) The main cellular IL-6 is located within the extracellular space and endoplasmic reticulum [Data source: Cellular compart¬ment database]. Table S1. Thermal cycler program for 894 G>T variant. Table S2. Correlations of IL-6 level with other parameters among HCC group. [file 43046_2024_238_MOESM1_ESM.docx]

| **(a)** 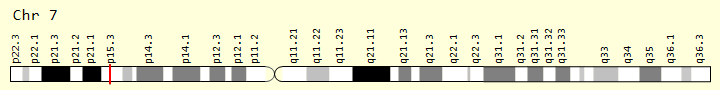 | |
| --- | --- |
| **(b)**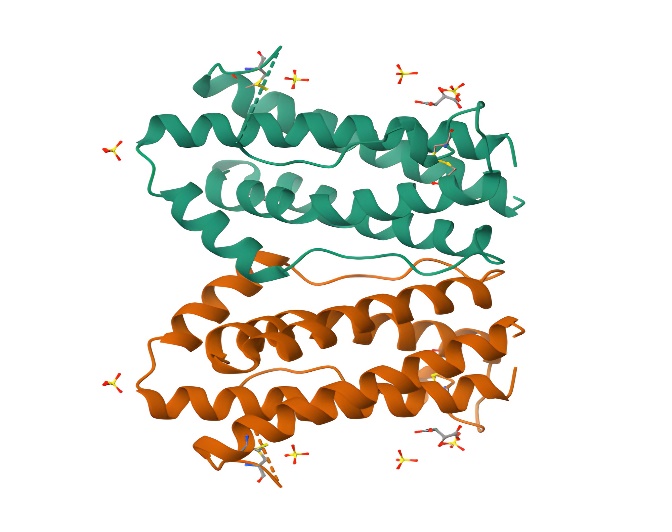 | **(c)**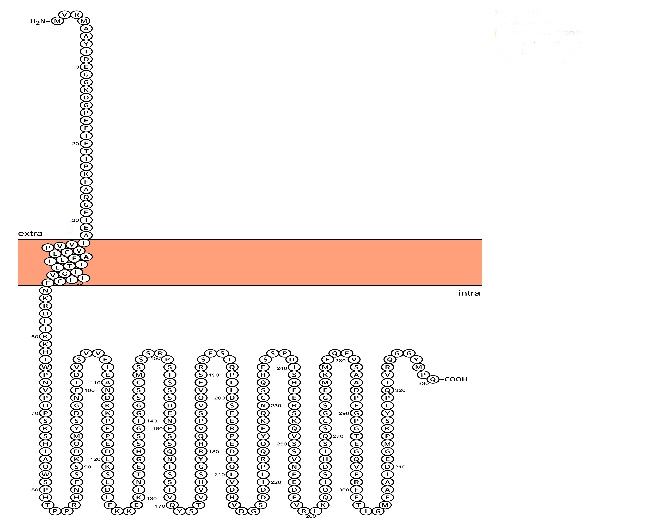 |
|  | |
| **(d)**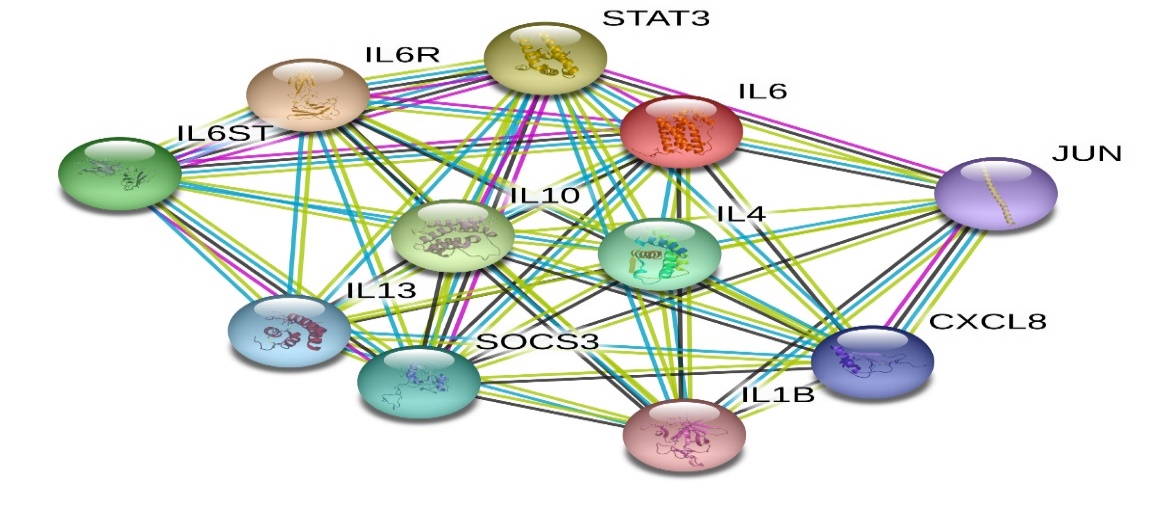 | |
| **(e)**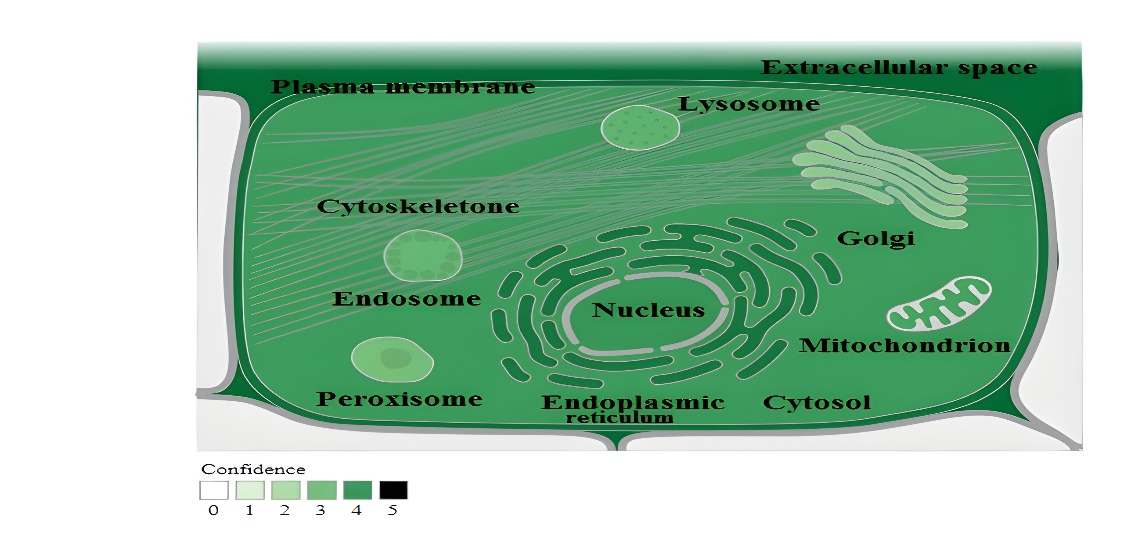 | |

**Fig. S1.** **(a)** The *IL-6* gene [[ENSG00000136244](http://www.ensembl.org/id/ENSG00000136244)] has some synonyms, including CDF, HGF, HSF, BSF2, BSF-2, IFNB2, and IFN-beta-2. The *IL-6*gene is located on the short arm of chromosome number 7 (Ch7p15.3). *IL-6*gene contains six exons and five introns. The (rs1800795) is an intronic variant guanine (C) to cytosine (G) replacement at −174 position (−174G/C) with the highest population MAF equals to 0.48 [Data source: Ensembl.org; Human Genome Assembly GRCh38.p13]. **(b, c)** The IL-6 gene encodes a signaling protein named interleukin-6 (IL-6) that comprises 212 amino acids with a molecular mass of 23,718 Daltons [Data source: Uniprot database (P05231)]. **(d)** Protein interaction networks recommend that IL-6 is a potent inducer of the acute phase response and has a major function in immune system regulation [Data source: STRING]. **(e)** The main cellular IL-6 is located within the extracellular space and endoplasmic reticulum [Data source: Cellular compart­ment database].

**Table S1**: **Thermal cycler program for *894 G>T* variant**

|  | **Stage** | **Temperature** | **Time** |
| --- | --- | --- | --- |
| 1 cycle | Initial denaturation | 94°C | 2 minutes |
| 30 cycles | Denaturation | 94°C | 30 seconds |
|  | Annealing | 54°C | 60 seconds |
|  | Extension | 72°C | 60 seconds |
| 1 cycle | Final extension | 72°C | 7 minutes |

**Table S2: Correlations of IL-6 level with other parameters among HCC group**.

| ***Parameter*** | ***r*** | ***p-value*** |
| --- | --- | --- |
| **Age** (years) | -0.007 | 0.93 |
| **ALT** (IU/L) | 0.07 | 0.42 |
| **AST** (IU/L) | 0.16 | 0.06 |
| **Total bilirubin** (mg/dl) | 0.035 | 0.69 |
| **Albumin** (g/dl) | 0.13 | 0.2 |
| **Creatinine** (mg/dl) | 0.1 | 0.26 |
| **AFP** (ng/ml) | 0.813 | <0.001* |
| **CRP** (mg/dl) | 0.981 | <0.001* |
| **RBCs** (× 1012/L) | -0.23 | 0.009 |
| **Hemoglobin** (g/dl) | -0.12 | 0.18 |
| **WBCs** (× 109/L) | 0.04 | 0.63 |
| **Platelets** (× 109/L) | -0.02 | 0.82 |

*: Significant at *p*˂0.05, r: correlation coefficient.
